# Mutation G61C in the *CRYGD* gene causing autosomal dominant congenital coralliform cataracts

**Published:** 2008-03-04

**Authors:** Feifeng Li, Shuzhen Wang, Chang Gao, Shiguo Liu, Baojian Zhao, Meng Zhang, Shangzhi Huang, Siquan Zhu, Xu Ma

**Affiliations:** 1 Graduate School, Peking Union Medical College; 2 Department of Genetics, National Research Institute for Family Planning; 3 Beijing Tongren Eye Center, Capital Medical University; 4 WHO Collaborative Center for Research in Human Reproduction, Beijing, China

## Abstract

**Purpose:**

We sought to identify the genetic defect in a four-generation Chinese family with autosomal dominant congenital coralliform cataracts and demonstrate the functional analysis of a candidate gene in the family.

**Methods:**

Family history data were recorded. Clinical and ophthalmologic examinations were performed on affected and unaffected family members. All the members were genotyped with microsatellite markers at loci considered to be associated with cataracts. Two-point LOD scores were calculated using the Linkage software after genotyping. A mutation was detected by direct sequencing, using gene-specific primers. Wild-type and mutant proteins were analyzed with online software.

**Results:**

Affected members of this family had coralliform cataracts. Linkage analysis was obtained at markers, D2S72 (LOD score [Z]=3.31, recombination fraction [θ]=0.0) and D2S1782 (Z=3.01, θ=0.0). Haplotype analysis indicated that the cataract gene was closely linked to these two markers. Sequencing the γD-crystallin gene (*CRYGD*) revealed a G>T transversion in exon 2, which caused a conservative substitution of Gly to Cys at codon 61 (P.G61C). This mutation co-segregated with the disease phenotype in all affected individuals and was not observed in any of the unaffected or 100 normal, unrelated individuals. Bioinformatic analyses showed that a highly conserved region was located around Gly61. Data generated with online software revealed that the mutation altered the protein’s stability, solvent-accessibility, and interactions with other proteins.

**Conclusions:**

This is the first reported case of a congenital coralliform cataract phenotype associated with the mutation of Gly61Cys (P.G61C) in the *CRYGD* gene; it demonstrates a possible mechanism of action for the mutant gene.

## Introduction

Hereditary congenital cataracts is a clinically and genetically heterogeneous lens disease responsible for a significant proportion of visual impairment and blindness in childhood [1,2] It can occur in an isolated fashion or as one component of a multi-system disorder. Non-syndromic congenital cataracts have an estimated incidence of 1–6 per 10,000 live births [3-6]; at least one-third of cases are familial.

From the first description of the cosegregation of inherited cataracts with the Duffy blood group locus [7], more than 30 loci have been mapped through linkage analysis and 17 genes have been characterized [8]. These include 10 genes encoding crystallins (*CRYAA, CRYAB, CRYBA1/A3, CRYBA, CRYBB1, CRYBB2, CRYBB3, CRYGC, CRYGD, CRYGS*), three genes encoding membrane transport proteins (*MIP, GJA3, GJA8*), one encoding a cytoskeletal protein (*BSFP2*), and three encoding transcription factors (*HSF4, MAF, PITX3*) [9]. The crystallin genes encode more than 90% of the water-soluble structural proteins present in the vertebrate crystallin lens and clearly represent compelling candidate genes for congenital cataracts.

Crystallins are subdivided into α-, β-, and γ-crystallins. γ- and β-crystallins are included in a superfamily of microbial stress proteins, which share a common two-domain structure, composed of four “Greek-key” motifs. They were thought to be specific to lens fiber cells, but it has been recently reported that some β- and γ-crystallin components were found in lens epithelial cells [10]. The unique spatial arrangement and solubility of the crystallins are essential to the optical transparency and high refractive index of the lens. Modification of the crystallins may disrupt their normal structure in the lens and cause cataracts [11].

Coralliform cataracts are an uncommon form of congenital cataract; it was first reported in 1895 [12] and subsequently described as an autosomal dominant trait in three British pedigrees circa 1910 [13]. Several studies [14-17] have shown that mutations in the *CRYGD* gene, located at 2q33–35, were responsible for coralliform, aceuliform, and fasciculiform phenotypes. To date, about 16 articles have reported *CRYGD* gene mutations that cause congenital cataracts [18-33] of which about five concern coralliform cataracts [21,23,27,33].

**Table 1:** Primer sequences used for sequencing *CRYGA, CRYGB, CRYGC*, and *CRYGD*.

**Gene (Exon)**	**Forward primers (5′→3′)**	**Reverse primers (5′→3′)**
*CRYGA* (1–2)	TCCCTTTTGTGTTGTTTTTGCC	TATGGCCATGGATCATTGATGC
*CRYGA* (3)	TCGTTGACACCCAAGGATGCATGC	TACAAGAGCCACTTAGTGCAGGG
*CRYGB* (1–2)	TGCAAATCCCCTACTCACCAAAATGG	TAAAAGATGGAAGGCAAAGACAGAGCC
*CRYGB* (3)	TAGGGACTGGAGCTTTAATTTCC	TACTAGTGCCAGAAACACAAGC
*CRYGC* (1–2)	TGCAGGATGTTAAGAGATGC	TTCTCTGATGTCCATCTAAGC
*CRYGC* (3)	TATTCCATGCCACAACCTACC	TTGACAGAAGTCAGCAATTGC
*CRYGD* (1–2)	TCTTTTGTGCGGTTCTTGCCAACG	TACCATCCAGTGAGTGTCCTGAGG
*CRYGD* (3)	TCTTTTTATTTCTGGGTCCGCC	TACAAGCAAATCAGTGCCAGG

We report a four-generation Chinese family with congenital coralliform cataracts. Linkage analysis mapped the disease gene to 2q33–35, and a missense mutation (181G→C) in *CRYGD* was identified in this family, resulting in the substitution of Gly61Cys (P.G61C) in *CRYGD*. Analysis of the wild-type and mutant proteins suggested that increased stability, complexity, and decreased hydrophilicity of the mutant protein may be the cause of coralliform congenital cataracts.

## Methods

### Clinical evaluation and DNA specimens

A four-generation family with non-syndromic congenital cataracts was recruited at the Beijing Tongren Eye Center, Capital Medical University, Beijing, China. Informed consent was obtained from each participant, consistent with the Declaration of Helsinki. Phenotype was documented by slit lamp photography. Genomic DNA was extracted from peripheral blood leukocytes using standard protocols.

**Figure 1:**
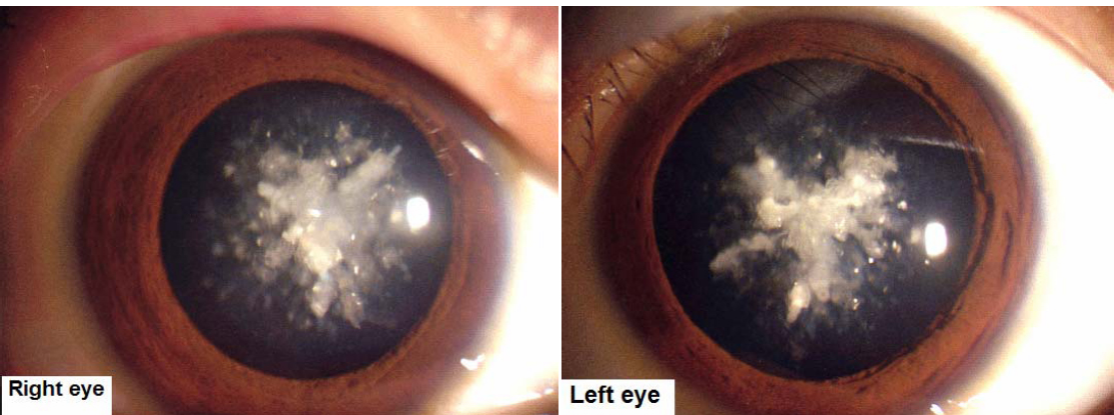
Slit lamp photographs of affected individual III:13. The photographs of the affected individual III:13 show that the opacities were coralliform cataract. The form of the opacification was irregular, similar to sea coral, with crystal clumps radiating from the center to the capsule.

### Genotyping

Polymerase chain reactions (PCRs) were performed with microsatellite markers close to candidate loci associated with autosomal congenital cataracts. PCR products from each DNA sample were separated on a 6% polyacrylamide gel and analyzed. Pedigree and haplotype data were managed using Cyrillic (version 2.1) software. Exclusion analysis was performed by allele sharing in affected individuals.

**Figure 2:**
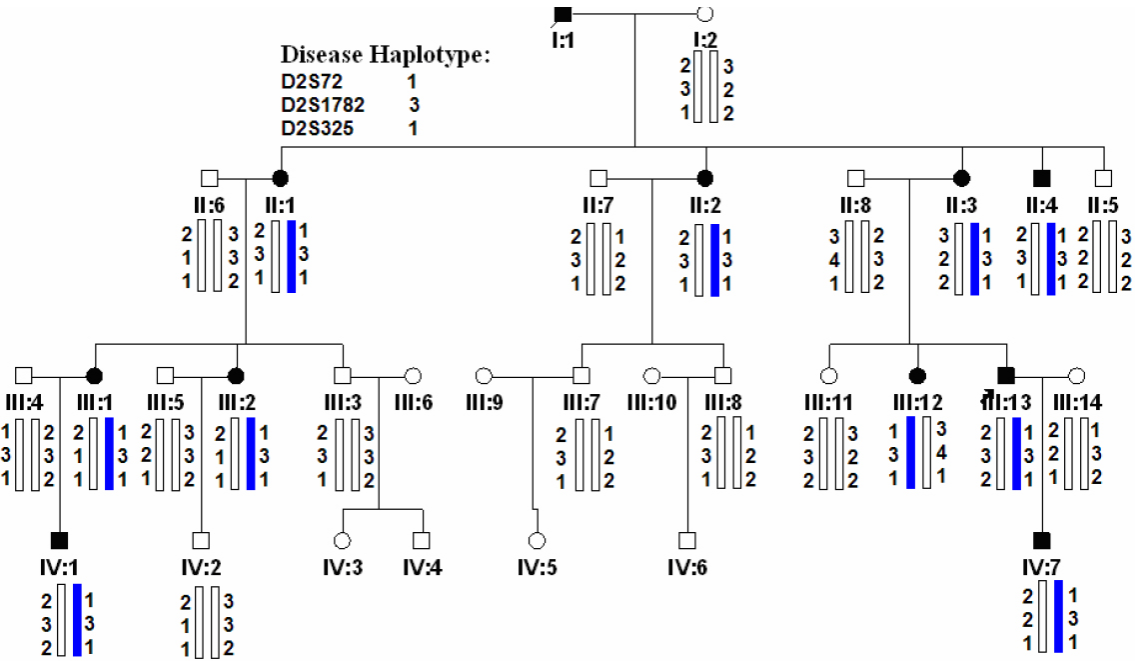
Pedigree and haplotype of the cataract family. A four-generation pedigree, segregating autosomal dominant coralliform cataract, is shown. Haplotyping shows segregation of two microsatellite markers on 2q. Squares and circles indicate males and females, respectively. Filled symbols and bars denote affected status.

### Linkage analysis

A two-point linkage was calculated with the LINKAGE (version 5.1) package. Autosomal dominant cataracts were analyzed with full penetrance and a gene frequency of 0.001. The allele frequencies for each marker were assumed to be equal in both genders. The marker order and distances between the markers were taken from the NCBI and GDB databases.

### DNA sequencing

Individual exons of the γ-crystallin gene cluster were amplified by PCR using primer pairs shown in Table 1 [34]. The PCR products were sequenced on an ABI3730 Automated Sequencer (PE Biosystems, Foster City, CA).

**Table 2:** Two-point LOD scores for linkage between cataract locus and markers on chromosome.

**Marker**	**LOD scores by recombination fraction (θ)**						
	0	0.04	0.09	0.14	0.19	0.24	0.29
D2S72	3.31	3.1	2.82	2.53	2.21	1.88	1.53
D2S1782	3.01	2.67	2.24	1.82	1.41	1.04	0.71
D2S325	1.81	1.65	1.44	1.23	1.01	0.78	0.5

### Denaturing HPLC

Denaturing HPLC was used to screen the mutation that was identified in the patients in the remaining patients, family members, and the 100 normal, unrelated control subjects in exon 2 of the *CRYGD* gene by using a commercial system (Wave DHPLC; Transgenomic, San Jose, CA).

### Computer construction and analysis of protein models

The tertiary structure of the protein is highly conserved. Both mutant and wild-type versions of the protein structure were predicted and analyzed using the Swiss-model software (version 3.5), CLC protein workbench 3 (version 3.0.2), and the Phyre software (version 0.2).

**Figure 3:**
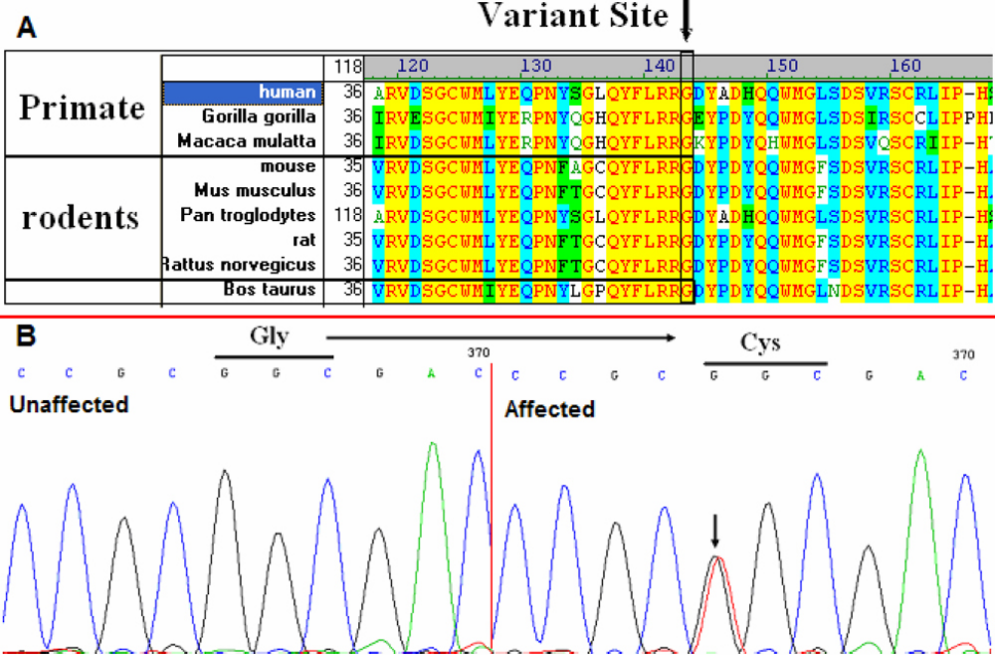
Multiple-sequence alignment and DNA sequence chromatograms of the *CRYGD* gene. **A**: Multiple-sequence alignment of *CRYGD* from primates, rodents, and cattle, to humans (Homo sapiens). The Gly61 residue is located within a highly conserved region. **B**: DNA sequence chromatograms of the P.G61C mutation in *CRYGD*. The G→T transversion at position 181 resulted in the P.G61C mutation.

Two-point LOD scores for chromosome 2q33–35 around the *CRYGD* locus. The highest observed LOD score was 3.31 (θ=0) for marker D2S72.

## Results

### Clinical data

The proband was a 26-year-old male (III: 13) who had bilateral cataracts from birth. The form of the opacification was irregular, similar to sea coral, with crystal clumps radiating from the center to the capsule (Figure 1). All affected individuals showed a phenotype of coralliform cataracts. This four-generation family included 11 affected individuals with congenital coralliform cataracts and 20 unaffected individuals (Figure 2). The diagnosis was confirmed by ophthalmologists. There was no history of other ocular or systemic abnormalities in the family.

### Linkage and haplotype analysis

The *CRYGD* gene on chromosome 2 was linked to this family while other candidate genes were excluded by allele sharing and linkage analysis. Significant linkage was found with markers D2S72 and D2S1782 and the maximum LOD score was 3.31 (at θ=0). Haplotype analysis showed that the responsible locus was localized at chromosome 2q33–35, flanked by markers D2S72, D2S325, and D2S1782 (Figure 2 and Table 2).

### Mutation analysis for *CRYGA*, *CRYGB*, *CRYGC*, and *CRYGD*

Direct cycle sequencing of the amplified fragments of *CRYGD* in two affected individuals identified a single base alteration, C.G181T (Figure 3B), in exon 2 of the *CRYGD* gene (GI: 181106), resulting in the substitution of Gly to Cys at codon 61 (P.G61C). The remainder of the coding sequence showed no other change.

### Multiple-sequence alignment and mutation analysis

From the NCBI and UCSC websites we obtained the *CRYGD* family protein-sequences and using the Vector NTI software, we obtained multiple-sequence alignments of *CRYGD* family proteins in various species, including primates, rodents and cattle (Figure 3A). We found that codon 61, where the mutation (P.G61C) occurred, was located in a highly conserved region of the protein.

**Figure 4:**
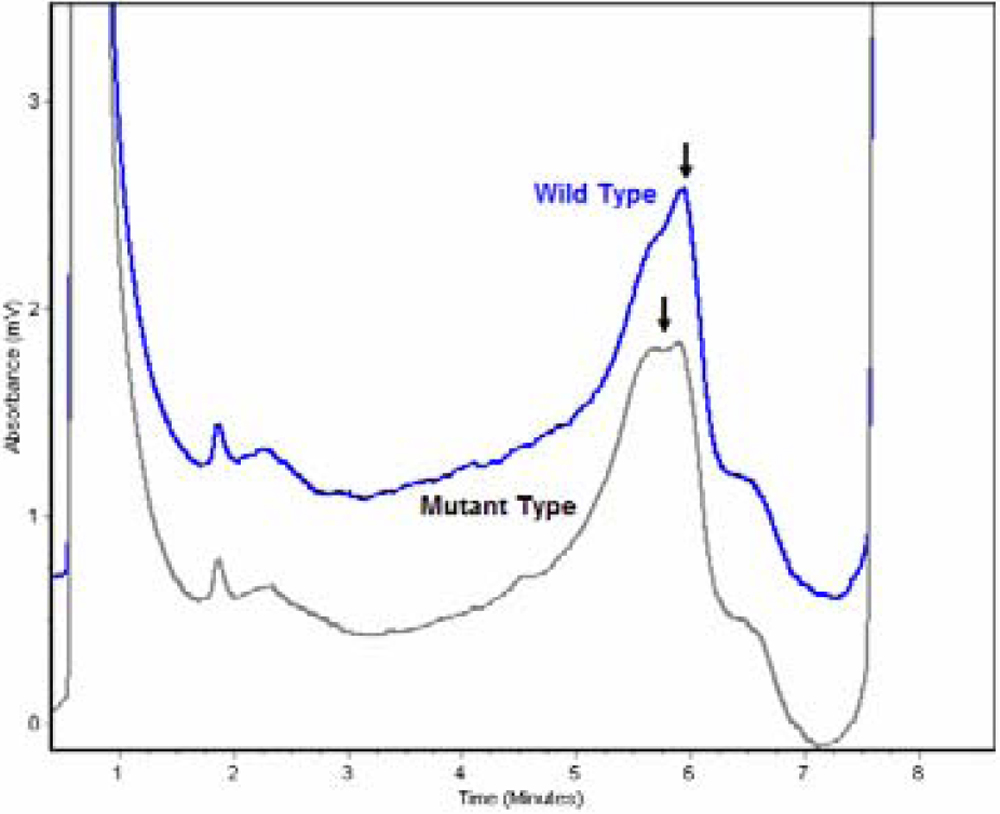
Denaturing high-performance liquid chromatography results of wild-type and mutated *CRYGD*. Denaturing HPLC results show variant traces for *CRYGD* compared to the wild-type (WT) trace. The profile in black is the mutant protein; the profile in blue is the wild-type protein.

### Denaturing HPLC

Denaturing HPLC analysis confirmed this mutation (Figure 4), which co-segregated with all affected individuals in the family. Further, this mutation was not observed in any of the unaffected family members or the 100 normal controls.

**Figure 5:**
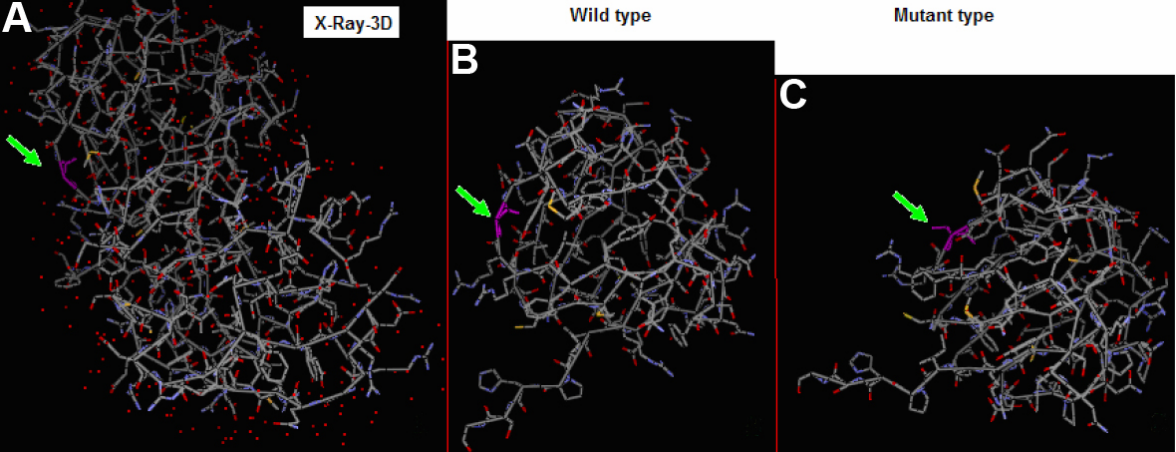
Comparison of wild-type and mutant CRYGD 3D-structures. **A**: The *CRYGD* 3D-structure is from PDB. Gly61 is indicated in pink. **B**: Wild-type *CRYGD* 3D-structure is shown using the Phyre software; Gly61 is indicated in pink. **C**: Mutant *CRYGD* 3D-structure is displayed using the Phyre software; Cys61 is indicated in pink. Comparing mutant and wild-type *CRYGD*, the 3D-structure did not significantly differ.

**Figure 6:**
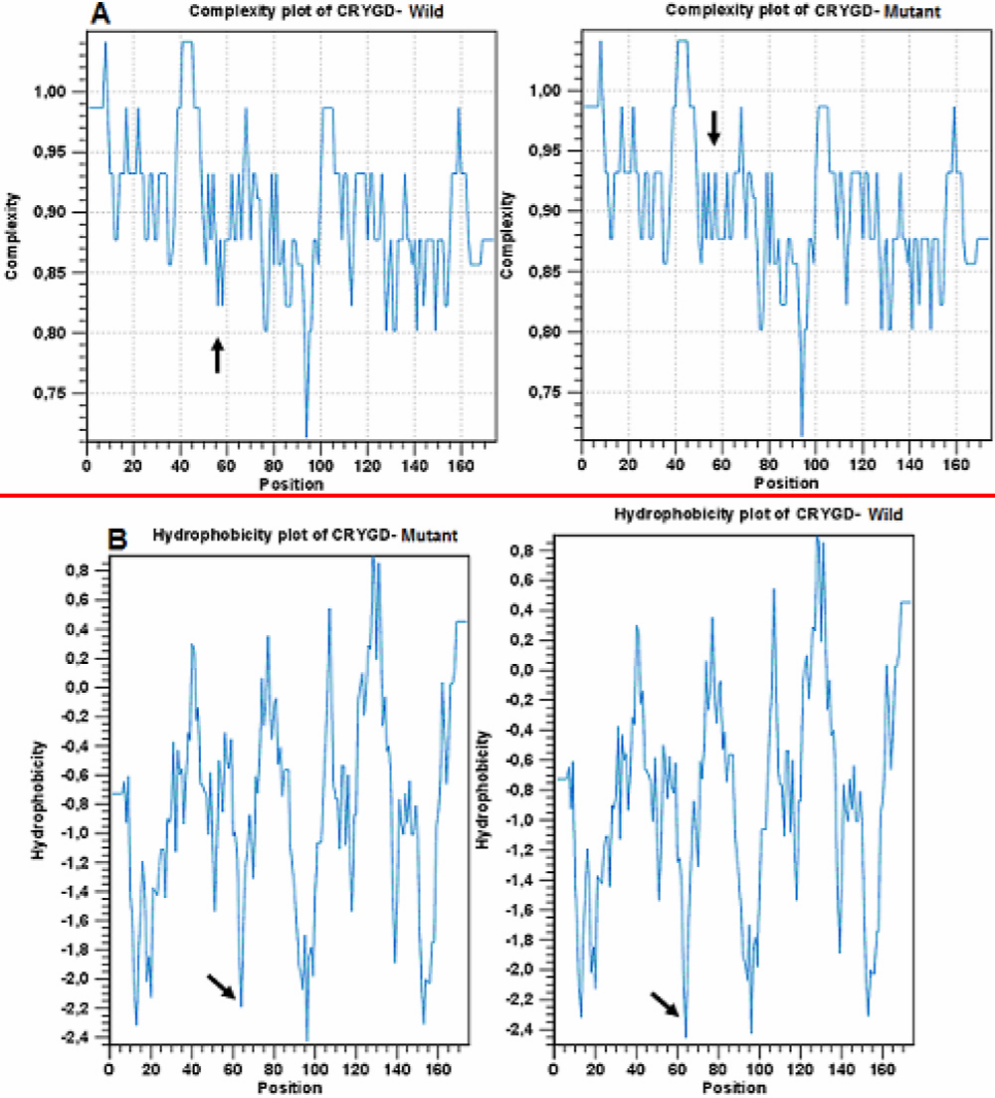
Comparison of complexity and hydrophobicity between wild-type and mutant CRYGD. CLC protein workbench 3 (version 3.0.2) predicted the effect of the substitution on *CRYGD* complexity (**A**) and hydrophobicity (**B**) of the protein. Complexity and hydrophobicity of the mutant protein increased (black arrow).

## Discussion

We identified a new mutation, P.G61C, in the *CRYGD* gene in a four-generation Chinese family with autosomal dominant congenital coralliform cataracts. The disease gene was linked to 2q33–35 with a maximum LOD score of 3.31, spanning the γD-crystallin gene cluster, which includes *CRYGA, CRYGB, CRYGC*, and *CRYGD*. Mutation analysis of the candidate gene detected a new mutation, P.G61C, in *CRYGD* that co-segregated with the disease phenotype in all affected individuals but was not present in the unaffected family members or 100 normal control subjects. The result of multiple-sequence alignments showed that Gly61 was a highly conserved residue.

**Figure 7:**
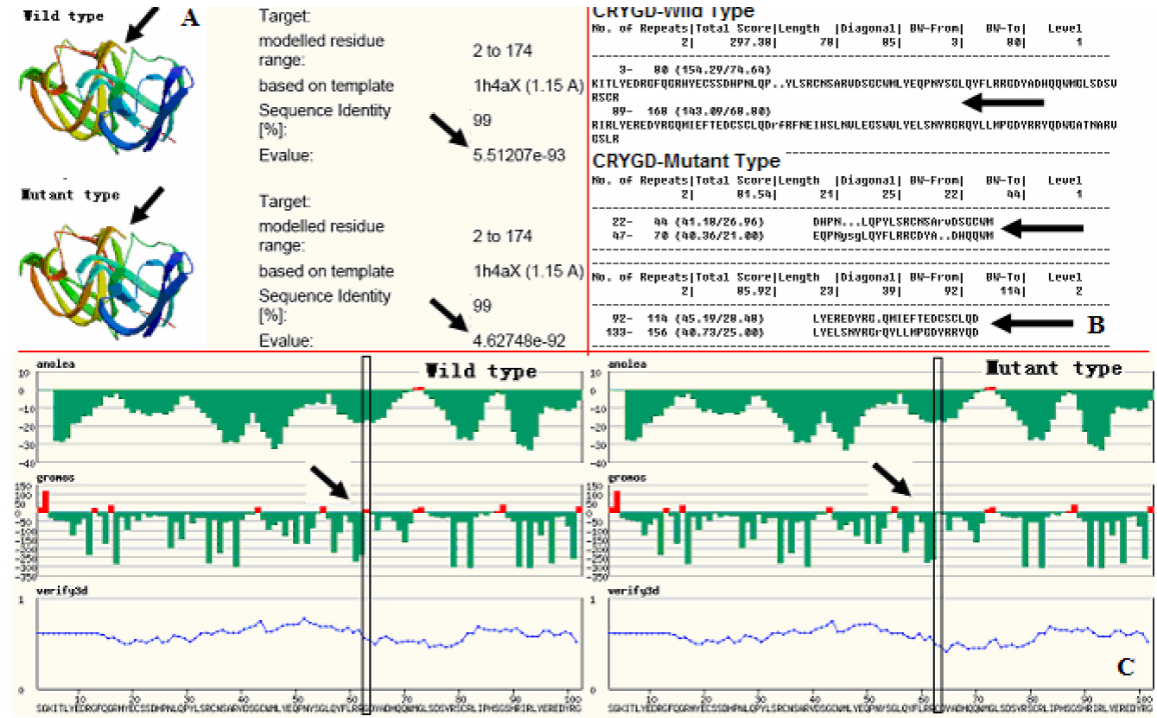
Using the online Radar software to predict the effect of the substitution in *CRYGD*. Effect on alignment of repeats in protein sequences is shown in **B**. The Swiss-model software (version 3.5) predicted that the P.G61C mutation exerted little effect on the tertiary structure of the protein but decreased the E-value and grooms value (**A**,**C**).

The lens crystallins constitute 80%–90% of the soluble proteins in the lens cells, and in most species, α-, β-, and γ-crystallins constitute the three main families. The human γ-crystallin gene cluster comprises six genes: *CRYGA, CRYGB, CRYGC, CRYGD, CRYGE*, and *CRYGF*, as well as a gene fragment of *CRYGG* [35]. In mammals, each of these genes consists of three exons; only *CRYGC* and *CRYGD* encode abundant lens γ-crystallins in humans [36,37]. *CRYGD* is one of the only two γ-crystallins to be expressed at high concentrations in the fiber cells of the embryonic human lens, which subsequently forms lens nucleus fibers [38-42]. For this reason and with the phenotype observed, we focused our attention on *CRYGD*. After screening for mutations in *CRYGA, CRYGB, CRYGC*, and *CRYGD* by direct cycle sequencing, we identified a G→T transversion in exon 2 of *GRYGD*, which was present only in affected members of the family. The transversion C.G181T located in exon 2 was predicted to cause a conservative substitution of Gly to Cys at codon 61 (P.G61C).

We used the online database PDB to study the three-dimensional (3D) structure of *CRYGD* (Figure 5A). This showed that Gly61 is an exposed surface residue on a strand. The online Phyre software (version 0.2) was used to compare the 3D-structures of the wild-type (Figure 5B) and mutant proteins (Figure 5C); the 3D-structure did not change much. CLC protein workbench 3 (version 3.0.2) predicted that the substitution in *CRYGD* would increase the complexity (Figure 6A) and hydrophobicity (Figure 6B) of the protein.

The online bioinformatics Swiss-model software (version 3.5) predicted both wild-type and mutant *CRYGD* structures; the P.G61C mutation exerted little effect on the tertiary structure of the protein but decreased the E-value and grooms value (Figure 7A,C). That is, the mutation is expected to stabilize the protein and affect the protein surface solvent accessibility and interactions with other proteins.

Furthermore, we used Radar to predict the effect that the substitution would have in the wild-type protein with an increase from one to two repeats (Figure 7B). Many large proteins have evolved by internal duplication, and many internal sequence repeats correspond to functional and structural units.

The alteration had little effect on the backbone or 3D-structure of the protein; complexity and hydrophobicity of the mutant protein increased while the E-value and grooms value decreased. It is known that γ-crystallin is one of three major lens crystallin components (α-, β-, and γ-crystallins) [43]. They form heterogeneous oligomers in the lens and have molecular weights ranging from 40 to 200 kDa [44]. The predicted new characteristics of the mutant protein, specifically decreased water solubility and increased stability of the oligomers, may be the cause of the disease.
